# Same-site transdifferentiation of basal cell carcinoma to squamous cell carcinoma as a mechanism of vismodegib treatment resistance: Two cases requiring multimodal and multidisciplinary limb-sparing techniques

**DOI:** 10.1016/j.jdcr.2023.12.001

**Published:** 2023-12-16

**Authors:** Brooke A. Burgess, Rebecca J. Wang, Joshua L. Owen

**Affiliations:** a Division of Dermatology, University of Texas Health Science Center San Antonio, San Antonio, Texas; b Long School of Medicine, University of Texas Health Science Center San Antonio, San Antonio, Texas; c Dermatology Service, South Texas Veterans Health Care System, San Antonio, Texas

**Keywords:** basal cell carcinoma, intralesional 5-fluorouracil, limb sparing, same-site squamous cell carcinoma, secondary malignancy, transdifferentiation, vismodegib

*To the Editor:* We found Zhao et al’s^1^ case report published October 2022 to be informative in describing metastatic same-site squamous cell carcinoma (SCC) arising during vismodegib therapy for basal cell carcinoma (BCC). We would like to supplement this report with observations of 2 patients with locally advanced upper limb BCC who were initially offered amputation, but who chose vismodegib treatment instead. They developed same-site SCC requiring multimodal and multidisciplinary approaches for limb salvage.

## Case 1

**Fig 1:**
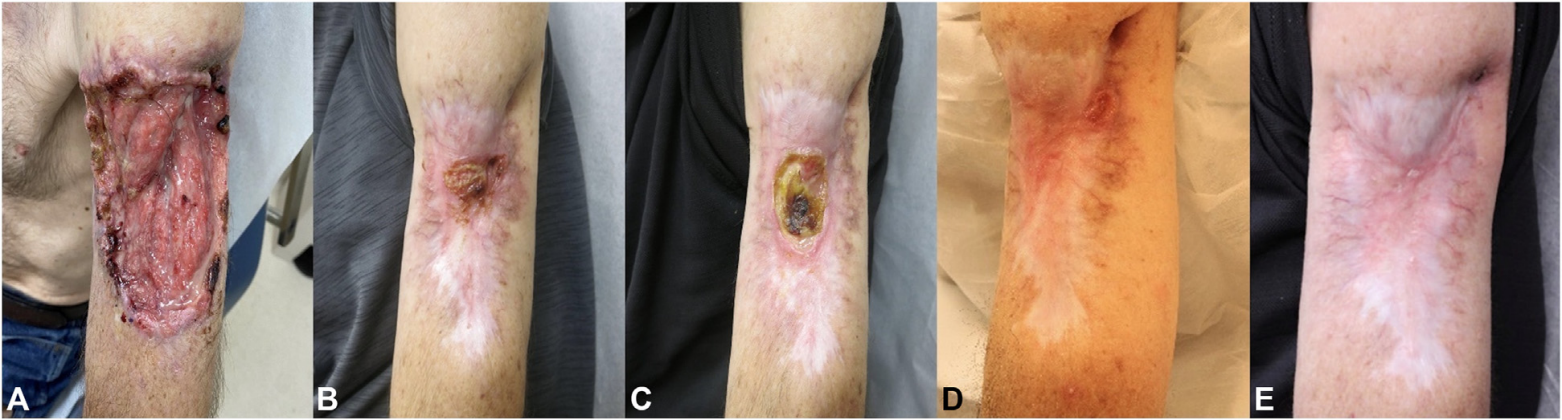
**A,** Case 1 on initial presentation with an 8 cm deep ulcerated lesion with visible muscle and peripheral rolled borders on the *left upper* arm (nodular basal cell carcinoma [*BCC*]). **B,** Moderately-differentiated infiltrating cystic squamous cell carcinoma in the *central* portion of the original BCC. **C,** Ulcer at site of squamous cell carcinoma after last injection of intralesional 5-fluorouracil. **D,** New ulcerated plaque with rolled borders to the *left* lateral proximal arm (nodular and ulcerated BCC). **E,** Clinical appearance after second intention healing after Mohs surgery. No clinical evidence of tumor was present.

A 61-year-old male presented with an 8-cm ulceration of the left upper arm with exposed musculature (Fig 1, *A*). Biopsy demonstrated nodular BCC, and magnetic resonance imaging (MRI) confirmed involvement of underlying musculature and humerus. The patient was offered limb amputation versus vismodegib therapy and elected for vismodegib. After 9 months of therapy with tumor shrinkage, a clinically resistant central tumor nodule persisted (Fig 1, *B*). Pathology was significant for moderately-differentiated infiltrating SCC. Vismodegib was continued; SCC was treated with 12 weekly intralesional 5-fluorouracil injections with clinical improvement (Fig 1, *C* and *D*). Three months after the final 5-fluorouracil injection, a new ulcerated tumor appeared in a separate area of the original tumor bed (Fig 1, *D*); pathology demonstrated nodular BCC. It was treated with Mohs micrographic surgery and vismodegib was discontinued. Two months later, despite no clinical evidence of tumor (Fig 1, *E*), follow-up MRI demonstrated multifocal, deep subcutaneous, and bony tumor involvement. Orthopedic surgery resected the involved soft tissue and partial thickness humerus; pathology showed moderately-differentiated SCC with focal areas of poor differentiation. Otolaryngology (facial plastics) repaired the site with a fibula free flap. The patient is currently undergoing radiotherapy and continues to experience good range of motion and strength 3 months postoperatively.

## Case 2

**Fig 2:**
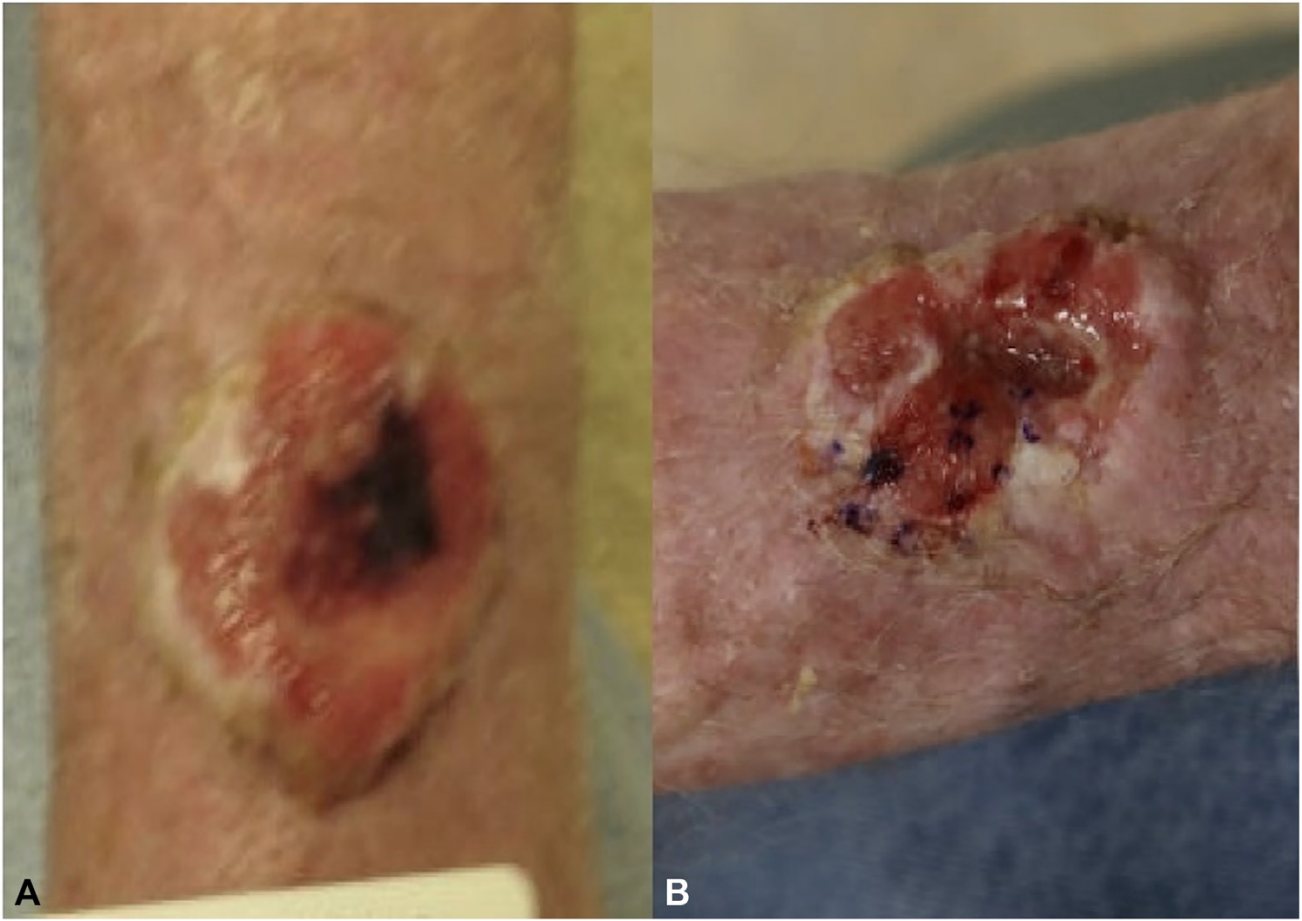
**A,** Case 2 on initial presentation with a 3 cm fungating, bleeding plaque to *left* dorsal forearm; pathology demonstrated nodular basal cell carcinoma with ulceration. **B,** New ulcerated plaque on *left* forearm; pathology showed squamous cell carcinoma.

A 65-year-old male presented with a 3-cm fungating plaque of the left dorsal forearm (Fig 2, *A*). Biopsy demonstrated nodular BCC; MRI revealed infiltration of the muscular fascia. The patient declined surgical intervention and was started on vismodegib. After 3.5 months of therapy, the patient self-discontinued after reduction in tumor size. Seven months after discontinuation, the tumor had enlarged. Repeat MRI demonstrated progressive involvement of underlying musculature. Vismodegib was reinitiated, and after 1 month of therapy, repeat biopsy of the tumor revealed SCC (Fig 2, *B*). Given the depth of tumor involvement, wide local excision was performed under general anesthesia by surgical oncology and plastic surgery followed by adjuvant radiotherapy. The patient has retained good range of motion and strength 15 months postoperatively.

Our cases demonstrate clinical challenges in treating BCC with vismodegib: resistance (BCC progression) and transdifferentiation (SCC development). Both patients were faced with potential limb amputation given the extent of local disease. Multimodal and multidisciplinary approaches were necessary to not only spare the involved limb but preserve its functionality. Both patients have retained full use of the involved limb, allowing for maintained quality of life.

If recalcitrant disease is present after/during vismodegib therapy, physicians should be aware of nonsurgical/surgical modalities or combinations thereof that might be efficacious; involving colleagues in other specialties should also be considered.

## Conflicts of interest

None disclosed.
